# Comorbidity of diarrhea and respiratory infection symptoms, and associated factors among under-five children in Gondar City, Northwest Ethiopia: a community-based cross-sectional study

**DOI:** 10.1186/s13052-025-01866-3

**Published:** 2025-02-24

**Authors:** Lidetu Demoze, Awrajaw Dessie, Jember Azanaw, Gelila Yitageasu, Kidist Asrat, Zemichael Gizaw

**Affiliations:** Department of Environmental and Occupational Health and Safety, Institute of Public Health, College of Medicine and Health Sciences, University of Gondar, Gondar, Ethiopia

**Keywords:** Comorbidity, Diarrhea, Respiratory infection symptoms, Gondar, Ethiopia

## Abstract

**Background:**

Childhood morbidity is frequently characterized by more than one health condition. Children under the age of five in low- and middle-income countries including Ethiopia experience multiple episodes of diarrhea and respiratory infection symptoms. However, there have been limited studies on comorbidities of diarrhea and respiratory infection symptoms. In addition, most studies conducted in Ethiopia seek separate outcomes for diarrhea and respiratory infection symptoms. Therefore, this study aimed to determine the prevalence of diarrhea and respiratory infection symptoms comorbidity, and associated factors among under-five children in Gondar City.

**Methods:**

Community-based cross-sectional study was conducted in Gondar City among under-five children from April 05 – May 04, 2023. Multi-stage sampling technique was used to collect a sample of 836. A structured questionnaire was employed through an interview-administered method for data collection at participants’ homes. Bivariable and multivariable binary logistic regression analyses were undertaken to identify predictors of childhood comorbidity of diarrhea and respiratory infection symptoms.

**Results:**

The comorbidity prevalence of diarrhea and respiratory infection symptoms in under-five children was 17.22% [CI: 14.8%-19.9%]. Mothers/caretaker age < 25 years (AOR = 3.52 at 95% CI:1.64,7.5), mothers/caretakers who had no formal education (AOR = 4.42 at 95% CI: 2.08,9.9.40), family size > 5 (AOR = 4.52 at 95% CI: 2.13,9.61), second birth order (AOR = 2.67 at 95% CI: 1.31,5.41), children playground not clean(AOR = 2.19 at 95% CI:1.01,4.71), started supplementary feeding at age > 6 months (AOR = 4.51 at 95% CI:1.50,13.58), mothers/caretakers who didn’t wash their hands after visiting latrine (AOR = 2.03 at 95% CI: 1.03,4.03), mothers/caretakers who didn’t wash their hands with soap and water (AOR = 1.92 at 95% CI: 1.00,3.69) were significantly associated factors with under five children comorbidity of diarrhea and respiratory infection symptoms.

**Conclusions:**

According to the findings, the prevalence of diarrhea and respiratory infection symptoms comorbidity was higher and variation in the amount of comorbidity is explained by maternal and child predictors. Educating mothers/caregivers about hand washing, sanitation, hygiene, and supplementary feeding is a key approach for the prevention and control of comorbidities in children.

## Background

Childhood morbidity is frequently characterized by more than one health condition [1]. Globally, pneumonia which is the main complication of Acute respiratory infection (14%) and diarrhea (14%) kills more children under the age of five than Human Immunodeficiency Virus (4%), malaria (16%), and measles (1%), combined [2]. Around 40 and 60 percent of children worldwide receive adequate treatment for the symptoms of diarrhea and acute respiratory infections (ARIs), respectively; nonetheless, diarrhea and ARI continue to be the leading causes of death for under five children [3]. According to World Health Organization reports, each year diarrhea takes the lives of more than 525,000 children under five years worldwide [4]. According to a systematic analysis conducted in 2015, approximately 4.4 million children under the age of five will die from infectious diseases such as diarrhea and ARI by 2030, with Sub-Saharan Africa accounting for 60% of these deaths [5]. In addition around 3 billion people use biomass [6] which is attributed to Indoor air pollution causing 1.5 to 2 million deaths per year worldwide, with 1 million occurring in children under the age of five due to ARI [7].

Acute respiratory infections in Sub-Saharan Africa, account for 42% of child fatalities [8] exposure to indoor air pollution during early childhood, impairs lung function and aggravates pre-existing conditions such as asthma [9]. Ethiopian Demographic and Health Survey 2016 found a 4.3 per cent childhood comorbidity of diarrhea and ARI among 9917 under-five children and residence, vaccination, and mother’s education were factors associated with comorbidity [10]. 50,320 infant deaths each year in Ethiopia due to IAP which represents 4.9% of the country’s overall illness burden [11]. EDHS 2016 report says 12% of under-five children experienced diarrhea in the two weeks preceding the survey [12]. Malnutrition and failure to thrive are also well-known risk factors for ARI therefore a causal link between diarrhea and subsequent risk of ARI is biologically plausible [13]. Confections of diarrhea and ARI were extremely common among children under five years of age seeking care due to overlapping risk factors such as poor indoor air environment, inadequate provision of water, hygiene, sanitation, overcrowding, breastfeeding for the first 6 months, socioeconomic status, the type of house the child lived in, the maternal occupation, are common risk factors for two diseases [2, 14]. Due to this factors comorbidity of diarrhea and respiratory infection symptoms diseases are common in under-five children in Africa and specifically, in Ethiopia. Therefore, this study provides insights for healthcare providers, highlighting the specific risks associated with ignoring comorbidities, which can lead to correct prioritization of public health interventions. There is limited evidence on the comorbidity of these two conditions, as most studies examine each condition separately. Accordingly, this study aimed to identify the prevalence of diarrhea and respiratory infection symptoms comorbidity, and associated factors among children under five children in Gondar City, Ethiopia, 2023.

## Methods

### Study design and setting

**Fig. 1:**
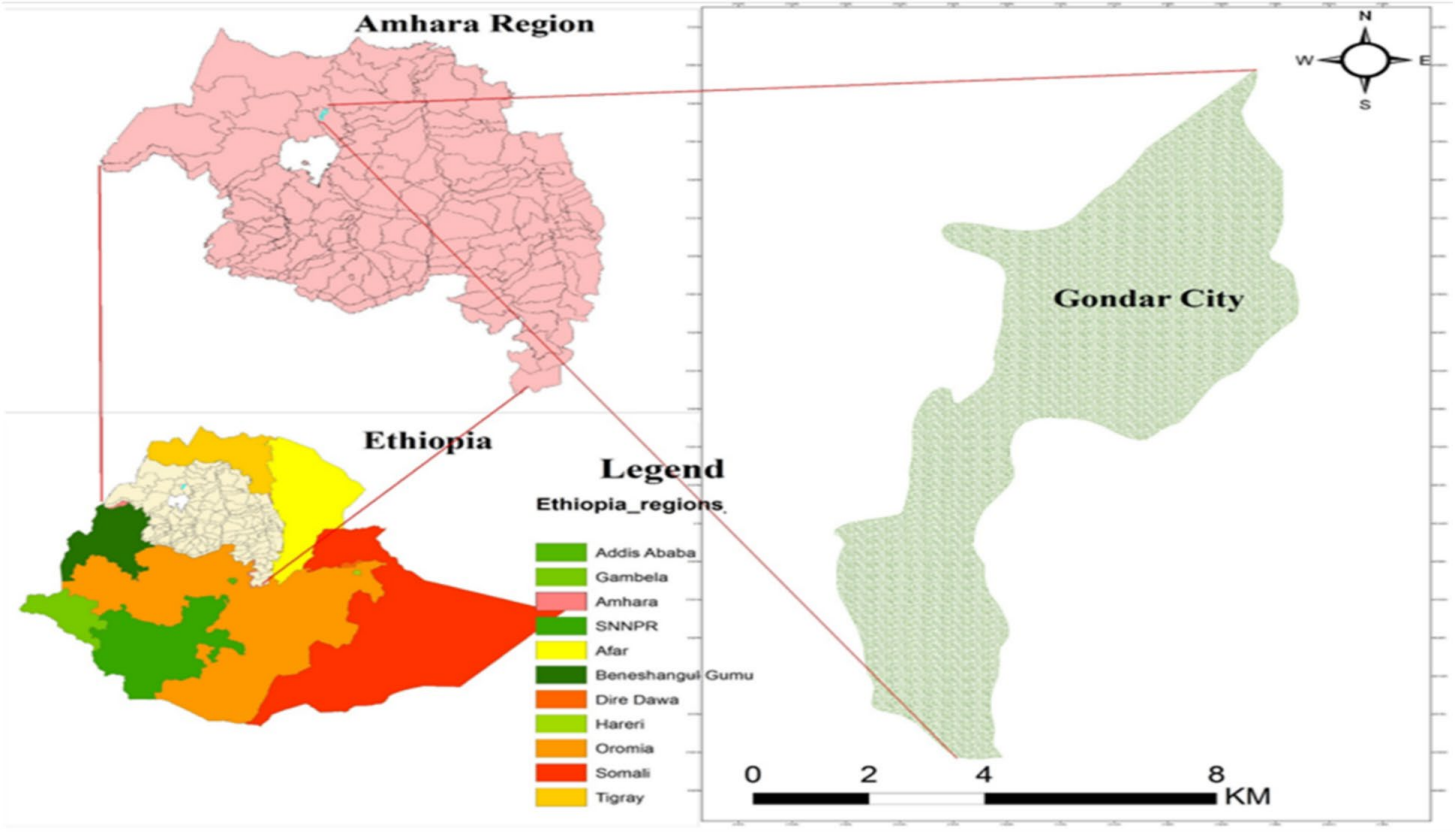
Map of the Gondar City, Northwest Ethiopia

A community-based cross-sectional study was conducted in Gondar City among under-five children between April 05 – May 04, 2023. Gondar City is approximately 734.3 km, from Addis Ababa and about 180 km from Bahir Dar City the capital of the Amhara region [15, 16]. According to the most recent administration report, Gondar has an estimated population of more than 454,446, with 218,378 men and 236,068 women. In the City, there are a total of 41,623 under-five children 20,191 males and 21,432 females. It has six sub-City administration areas comprised of 36 kebeles. The City has nine health centers, one referral hospital, and one general hospital that serves the people of Gondar City and the surrounding area (Fig. 1).

### Sample size calculation and sampling procedures

The sample size was calculated using a single population proportion formula while keeping the following assumptions in mind: P = 50% of children with comorbidity of diarrhea and respiratory infection symptoms and (no previous study in the study area at the time of the study), 95% confidence interval, 5% margin of error (d), and design effect 2.

$$n\;=\frac{\left({\displaystyle\frac{Z\alpha}2}\right)^2\;P(1-P)}{d^2}\;=\;\frac{\left(1.96\right)^{2\ast}0.5^\ast0.5}{0.05^2}\;=384$$

By taking 10% of the non-response rate, then the total sample size was 845.

**Fig. 2:**
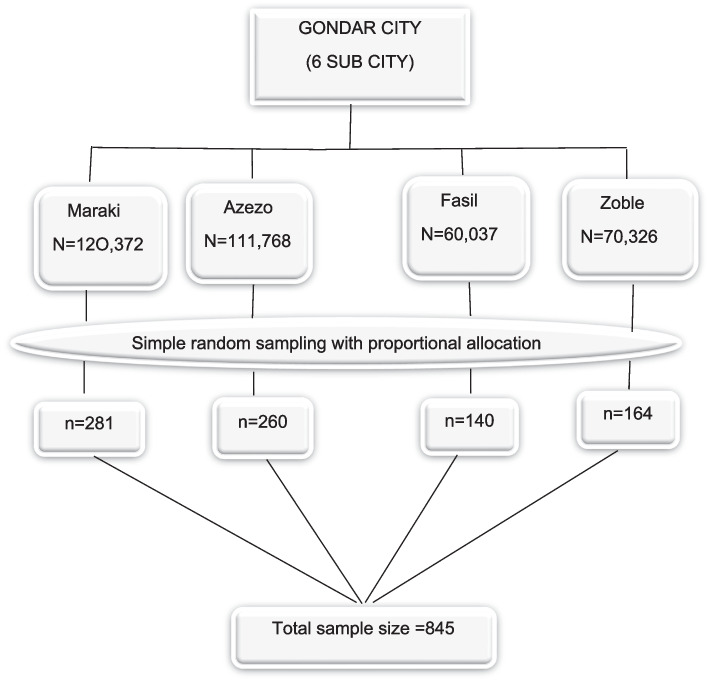
A Flow chart of the sampling procedures for the selection of study participants in Gondar City, northwest Ethiopia, 2023

The target populations were included in the study using multi-stage sampling technique with a total of two stages (Fig. 2).

### Data collection tools and procedures

Structured and pretested questionnaires were used to collect data. The questionnaire was prepared based on a review of relevant literature [17–20]. The questionnaire was first prepared in English language and translated to the local Amharic language, and back-translated into English to check consistency. Data were collected from mothers/primary caretakers using an interviewer-administered method. The questionnaire includes a total of four parts which include the socio-demographic characteristics, the child’s diarrhea and respiratory infection symptoms, environmental, household and behavioral factors respectively. Data collectors were given two-day training on the tool & exercise it. The questionnaire discussed thoroughly question by question. The data collection process and completeness of data were closely supervised.

### Measurement of study variables

Comorbidity was defined as the mother/caretaker self-reported that a child had the occurrence of diarrhea and respiratory infection symptoms together or in a sequential manner [2]. Respiratory infection symptoms were defined as the mother/caretaker self-reported the child had been suffering from cough, shortness of breath, wheezing, phlegm and blocked or running nose in the past two weeks [21]. Diarrhea was defined as the mother/caretaker self-reported that the child had three or more loose or watery stools in 24 h, in the past two weeks before data collection [22].

### Data processing and analysis

All the questionnaires were checked manually for completeness, coded, and entered into EPI info version 7.1.5.2 and exported to Stata version 14.1 software for further analyses. Descriptive analyses were done to describe variables using summary measures, frequencies, figures & tables. Comorbidity of diarrhea and respiratory infection symptoms evaluated by running binary logistic regression. Then explanatory variables with a *P*-value < 0.20 in bivariable logistic regression were analyzed in multivariable regression. The degree of association between outcome & explanatory variables was assessed using odds ratios and a 95% confidence interval.

Independent Variables with a *p*-value < 0.05 in multivariable regression are considered statistically significant. Finally, the multi-collinearity of variables was assessed by calculating the Variance Inflation Factor (VIF). Additionally, the Goodness of fit of the model was checked by Hosmer and Lemeshow.

## Results

### Socio-demographic characteristics of mothers/caretakers

A total of 836 participants were enrolled in this study, with a response rate of 98.93%. Seven hundred seventy-nine (93.18%) were female caretakers and fifty-seven were male caretakers (6. 82%), the median age is 29. The majority (87.44%) of study participants were Orthodox Christian in religion followed by Muslims (9.69%). Five hundred eighteen (61.96%) of caretakers were housewives (Table 1).

**Table 1:** Socio-demographic characteristics of mothers/caretakers in Gondar City between March –April 2023(*n* = 836)

**Variable **	**Category**	** Frequency(n)**	**Percent(%)**
**Sex **	Female	779	93.18
	Male	57	6.82
**Age **	<25	191	22.85
	25-27	193	23.09
	28-31	152	18.18
	≥32	300	35.89
**The median age of the mother/caretaker is 29 ± 7.63(SD)**			
**Religion **	Orthodox	731	87.44
	Muslim	81	9.69
	Other*	24	2.87
**Marital status **	Married	719	86.00
	Divorced	46	5.50
	Other **	71	8.50
**Educational status **	No	123	14.71
	Primary	263	31.46
	Secondary and above	450	53.83
**Education status of the spouse**	No	91	11.65
	Primary	216	27.66
	Secondary and above	474	60.69
**Average monthly ** **Income(Ethiopian Birr)**	1000-4876	213	25.48
	4877-5643	206	24.64
	5644-8000	243	29.07
	≥8001	174	20.81
**Occupation of mother **	Housewife	518	61.96
	Farmer	8	0.96
	Student	24	2.87
	Private	112	13.40
	Government	132	15.79
	Merchant	31	3.71
	Other***	11	1.32
**Occupation of spouse**	Farmer	11	1.42
	Student	7	0.90
	Private	486	62.71
	Government	190	24.52
	Merchant	53	6.84
	Other***	28	3.61
**House ownership**	Private/owned	229	27.39
	Rent from kebele	71	8.49
	Rent from private	530	63.40
	Neither	6	0.72
**Family size **	≤5 persons	638	76.32
	> 5 persons	198	23.68
**Relation of the Respondent to the child**	Mother	748	89.47
	Caretaker	88	10.53
**Number of rooms **	One	513	61.36
	Two	191	22.85
	Three and above	132	15.79
**Separate bedroom**	No	607	72.61
	Yes	229	27.39
**Separate kitchen **	No	598	71.53
	Yes	238	28.47

Others* = Protestant, Jewish, or Catholic

Other**=single, separated, widowed

Others*** = daily laborer, driver, or priest

### Child-related socio-demographic factors

Of the 836 under five children, the majority, 255 (30.50%), were between 48 and 59 months old, while 243 (29.07%) were between 24 and 35 months. Four hundred thirty (51.44%) were males and four hundred six (48.56%) were females (Table 2).

**Table 2:** Child-related socio-demographic factors in Gondar City between March–April 2023(*n* = 836)

Variable	Category	Frequency(n)	Percent (%)
**Age in month**	< 12	87	10.41
	12–23	79	9.45
	24–35	243	29.07
	36–47	172	20.57
	48–59	255	30.50
**Sex**	Male	430	51.44
	Female	406	48.56
**Birth order of a child**	First	330	39.47
	Second	269	32.18
	Third	112	13.40
	Fourth	81	9.69
	Fifth and above	44	5.26
**Number of under-five children in the household**	One	571	68.30
	Two	246	29.43
	Three or more	19	2.27

### Housing characteristics and latrine facility status

Only a few households (6.94%) have kitchen exhaust and the majority of the households six hundred seventy-one (80.26%) constructed their house with mud and less than one-fifth (19.14%) constructed their house with the concert. More than three-fourths (88.28%) of the households have latrine facilities from this majority of facilities were pit latrines with slab (79.89%) and (85.77%) households have shared latrine facilities and less than one-fifth (18.78%) of the household have hand washing facilities. (79.19%) household practice cooking inside the house (Table 3).

**Table 3:** Housing characteristics and latrine facility status in Gondar City between March –April 2023(*n* = 836)

Variable	Category	Frequency(n)	Percent (%)
**Kitchen exhaust**	No	778	93.06
	Yes	58	6.94
**No functional windows**	None	80	9.57
	One	564	67.46
	Two	147	17.58
	≥ Three	45	5.38
**Floor construction mud**	No	165	19.74
	Yes	671	80.26
**Floor construction materials concrete**	No	676	80.86
	Yes	160	19.14
**Floor construction brick**	No	815	97.49
	Yes	21	2.51
**Wall surface water base paint**	No	340	40.67
	Yes	496	59.33
**Ceiling surface**	Wooden	662	79.19
	Painted	174	20.81
**Damp stains**	No	278	33.25
	Yes	558	66.75
**Visible mold**	No	351	41.99
	Yes	485	58.01
**Cooking**	Inside	662	79.19
	Outside	174	20.81
**Hand washing facility**	No	679	81.22
	Yes	157	18.78
**Latrine**	No	98	11.72
	Yes	738	88.28
**Type of latrine**	Flush to septic tank	51	6.93
	Flush to pit latrine	25	3.40
	VIP	71	9.65
	Pit with slab	581	78.94
	Pit without slab	8	1.09
**The proximity of the latrine from home**	1–9 m	550	74.46
	≥ 10 m	188	25.54
**Faeces around the pit hole**	No	213	28.92
	Yes	525	71.08
**Faeces around the compound**	No	527	63.04
	Yes	309	36.96
**If the household has no latrine where do they dispose of the human waste**	Open field	86	87.76
	Other*	12	12.24
**Ownership of latrine**	Private	105	14.23
	Shared	633	85.77
**Child playground**	Not clean	615	73.56
	Clean	221	26.44

Other* = Buried underground, plastic bags

### Waste disposal and water-related characteristics of the respondents

More than half (58.37%) of household uses a garbage can for waste disposal and eight hundred (95.69%) have improved water source (Table 4).

**Table 4:** Waste disposal and water-related characteristics in Gondar City between March –April 2023(*n* = 836)

Variable	Category	Frequency(n)	Percent (%)
**Waste disposal**	Pit	14	1.67
	Open	268	32.06
	Burning	49	5.86
	Garbage	488	58.37
	Other*	17	2.03
**Waste Collection container**	Plastic	785	93.90
	Iron	2	0.24
	Jerry can	40	4.78
	Other**	9	1.08
**Source of water supply**	Improved	800	95.69
	Unimproved	36	4.31
**Daily requirements of water**	< 20 litters	52	6.22
	≥ 20 litters	784	93.78
**How long it takes to reach the water source(round trip)**	< 15 min	765	91.51
	≥ 15 min	71	8.49

Other* = Dispose into the river

Other** = pot

### Childhood diarrheal diseases and respiratory infection symptoms comorbidity in Gondar City

The prevalence of diarrhea among under-five children was 24.64% and the prevalence of respiratory infection symptoms among under-five children was 35.29%.

### The comorbidity prevalence of diarrhea and respiratory infection symptoms among under-five children

**Fig. 3:**
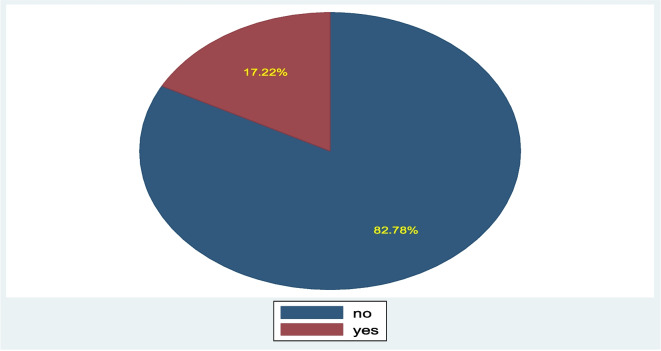
The comorbidity prevalence of diarrhea and respiratory infection symptoms (cough, phlegm, shortness of breath, wheezing and runny nose) among under-five children in Gondar City, northwest Ethiopia, 2023

The comorbidity prevalence of diarrhea and respiratory infection symptoms at a 95% confidence interval was 17.22% [CI: 14.8%-19.9%] (Fig. 3).

### Determinants of comorbidity among under-five children

On bivariable analysis, the age of the mother/caretakers, educational status of mother/caretaker, educational status of spouse, average monthly income, family size, number of rooms, birth order, separate bedroom, child sex, kitchen cleanse, a father with asthma, charcoal, smoking in the building, cleaning frequency, hand washing facility, kitchen floor, flies observed around the latrine facilities, refuse disposal, child playground, uncollected garbage, exposure to animal allergens in past, supplementary feeding, breastfeeding status, feed powder milk, feeding with hand, wash hand before food preparation and eating, wash hand after feeding child, wash hand after visiting latrine, wash with soap and water and wash hand with only water were significantly associated with comorbidity of diarrhea and respiratory infection symptoms.

Finally, on multivariable analysis, the age of mothers/caretakers < 25 years, educational status of mothers/caretakers, birth order, family size, child playground, supplementary feeding, hand washing with soap and water, and hand washing after visiting latrine were significantly associated with under-five children’s comorbidity of diarrhea and respiratory infection symptoms.

Under-five children whose mothers/caretakers age < 25 years were 3.52 times more likely to develop comorbidity than those whose age was higher and more mature (AOR = 3.52 at 95% CI: 1.64, 7.55). Under-five children whose mothers/caretakers had no education were 4.42 times more likely to encounter comorbidity than those who enrolled in secondary and above formal educations (AOR = 4.42 at 95% CI: 2.08, 9.40). Households with family size greater than five were 4.52 times more likely to develop comorbidity of children under five years than their counterparts (AOR = 4.52 at 95% CI: 2.13,9.61). Under five children whose birth order is second were 2.67 times more likely to encounter comorbidity than those with first orders (AOR = 2.67 at 95% CI: 1.31, 5.41).

Under five children whose playground is not clean were 2.19 times more likely to develop comorbidity than their counterparts (AOR = 2.19 at 95% CI: 1.01, 4.71). Under-five children who started supplementary feeding at an age greater than 6 months were 4.51 times more likely to develop comorbidity than their under-five children who started supplementary feeding at 6 months and lower (AOR = 4.51 at 95% CI:1.50,13.58).

Under five children whose mothers/caretakers didn’t wash their hands after visiting latrine regular basis, their children were 2.03 times more likely to develop comorbidity than their counterparts (AOR = 2.03 at 95% CI: 1.03,4.03). Under five children whose mothers/caretakers didn’t wash their hands with soap and water their children were at 1.92 times higher risk of encountering comorbidity than those who wash their hands with soap and water regularly (AOR = 1.92 at 95% CI: 1.00,3.69) (Table 5).

## Bi-variable and multi-variable binary logistic regression analysis of associated factors with comorbidity

**Table 5:** Factors associated with under-five children comorbidity of diarrhea and respiratory infection symptoms in Gondar City northwest Ethiopia, 2023

		Child with Comorbid		COR (CI%95)	AOR (CI%95)
Variables	Categories	No	Yes		
Age of mother/caretaker	< 25 25–27 28–31 ≥ 32	132 170 126 264	59 23 26 36	3.27(2.06,5.21) 0.99(0.56,1.80) 1.51(0.87,2.61) 1	3.52(1.64,7.55) * 1.54(0.66,3.63) 2.03(0.94,4.38) 1
Educational status of mother/caretaker	No Primary Secondary and above	69 213 410	54 50 40	3.33(2.08,5.33) 1 0.41(0.26,0.65)	4.42(2.08,9.40)* 1 0.85(0.42,1.72)
Educational status of spouse	No Primary Secondary and above	72 168 408	19 48 66	0.92(0.50,1.68) 1 0.56(0.37,0.85)	0.87(0.34,2.19) 1 1.29(0.66,2.50)
Average monthly income (Ethiopian Birr)	1000–4876 4877–5643 5644–8000 ≥ 8001	166 156 213 157	47 50 30 17	2.61(1.44,4.74) 2.96(1.63,5.35) 1.30(0.69,2.44) 1	0.75(0.30,1.86) 1.74(0.72,4.16) 1.46(0.61,3.46) 1
Family size	≤ 5 > 5	557 135	81 63	1 3.20(2.19,4.68)	1 4.52(2.13,9.61) *
Number of under five	One Two Three and more	469 210 13	102 36 6	1 0.78(0.52,1.19) 2.12(0.79,5.71)	1 0.56(0.30,1.07) 1.51(0.33,6.82)
Number of room	One Two Three and above	413 161 118	100 30 14	1.29(.83,2.03) 1 0.63(0.32,1.25)	0.76(0.33,1.74) 1 1.42(0.52,3.92)
Birth order	First Second Third Fourth Fifth and above	284 207 93 69 39	46 62 19 12 5	1 1.84(1.21,2.81) 1.26(0.70,2.26) 1.07(0.53,2.13) 0.79(0.29,2.11)	1 2.67(1.31,5.41) * 1.23(0.46,3.31) 0.32(0.09,1.09) 0.25(0.05,1.14)
Separate bedroom	No Yes	493 299	114 30	1.53(0.99,2.36) 1	1.42(0.60,3.39) 1
Child sex	Male Female	347 345	83 61	1.35(0.94,1.94) 1	1.41(0.81,2.46) 1
Kitchen cleanse	Clean Not clean	488 204	69 75	1 2.60(1.80,3.74)	1 1.62(0.86,3.04)
Father with asthma	No Yes	665 27	132 12	1 2.23(1.10,4.53)	1 2.04(0.63,6.55)
Charcoal	No Yes	192 500	23 121	1 2.02(1.25,3.25)	1 1.06(0.46,2.46)
Smoking in the building	No Yes	637 55	115 29	1 2.92(1.78,4.77)	1 1.78(0.78,4.09)
Cleaning frequency	One time Two times Three times > 3 times	331 279 64 18	74 46 21 3	1.35(0.90,2.02) 1 1.99(1.11,3.56) 1.01(0.28,3.56)	1.03(0.53,2.02) 1 2.01(0.79,5.10) 1.72(0.24,12.3)
Hand washing facility	No Yes	552 140	127 17	1.89(1.10,3.24) 1	0.78(0.32,1.86) 1
Kitchen floor	Clay & mud Cement Other	576 104 12	122 15 7	1.34(0.76,2.36) 1 3.75(1.28,10.9)	0.83(0.32,2.12) 1 2.15(0.40,11.57)
Flies observed around the latrine facilities	No Yes	226 439	15 122	1 4.18(2.39,7.39)	1 2.15(0.95,4.86)
Refuse disposal	Pit Open Burning Garbage can Other	12 212 39 419 10	2 56 10 69 7	1 1.58(0.34,7.28) 1.53(0.29,8.01) 0.98(0.21,4.51) 4.2(0.70,24.94)	1 0.21(0.03,1.40) 0.26(0.03,2.19) 0.31(0.05,1.91) 0.54(0.05,5.38)
Child playground	Not clean Clean	484 208	131 13	4.33(2.39,7.83) 1	2.19(1.01,4.71) * 1
Uncollected garbage/solid waste	No Yes	179 513	11 133	1 4.21(2.22,7.98)	1 1.43(0.58,3.54)
Exposure to animal allergens in past	No Yes	641 51	126 18	1 1.79(1.01,3.17)	1 1.12(0.44,2.87)
Supplementary feeding	< 6 month = 6 month > 6 month	111 448 133	11 45 88	1 1.01(0.50,2.02) 6.67(3.39,13.1)	1 1.40(0.47,4.18) 4.51(1.50,13.58) *
Breastfeeding status	Exclusive Partial Not	21 214 457	5 65 74	1.47(0.53,4.02) 1.87(1.29,2.71) 1	1.87(0.32,10.75) 1.69(0.94,3.02) 1
Feeding the child powder milk	No Yes	551 141	126 18	1.79(1.05,3.03) 1	0.91(0.40,2.06) 1
Feeding the child with a hand	No Yes	160 532	12 132	1 3.30(1.78,6.13)	1 1.30(0.44,3.80)
Wash hands before food preparation and eating	No Yes	67 625	46 98	4.37(2.84,6.74) 1	1.86(0.80,4.29) 1
Wash hands after feeding the child	No Yes	143 549	42 102	1.58(1.05,2.36) 1	1.92(0.85,4.35) 1
Wash hands after visiting the latrine	No Yes	257 435	101 43	3.97(2.69,5.86) 1	2.03(1.03,4.03) * 1
Wash hands with soap and water	No Yes	136 556	61 83	3.00(2.05,4.39) 1	1.92(1.00,3.69) * 1
Wash hands with only water	No Yes	319 373	26 118	1 3.68(2.36,5.74)	1 1.74(0.95,3.21)

*AOR* adjusted odds ratio, *CI* confidence interval, *COR* crude odds ratio

Hosmer and Lemeshow test = 0.7502 showed that the model was fitted well

* = statistically significant at *p* < 0.05

* = statistically significant at *p* < 0.01

* = statistically significant at *p* < 0.001

### Discussion

A community-based cross-sectional study was conducted to determine the prevalence and factors associated with comorbidity of diarrhea and respiratory infection symptoms among under-five children in Gondar City. Children who experienced both diarrhea and respiratory infection symptoms have a prevalence of 17.22% which is higher than the previous study conducted in Ethiopia EDHS 4.6% [10] and Kenya DHS(2.2%) [2] children had comorbidity from diarrhea and ARI respectively.

Similarly, the prevalence was higher than Ghana and Congo DHS having a comorbidity of diarrhea and ARI was 11% and 3.9% consecutively [23, 24]. Similarly, Adedokun’s [25] Nigerian study found that 9% of under-five children had comorbidity. In addition, the prevalence was higher than the study conducted in Myanmar which was 3.7% [26]. Our finding was comparable with a scoping review from 6 studies stating that the prevalence of multimorbidity among under-five children in sub-Saharan African countries ranged from 1.2% to 24.8%.

The overall prevalence in our finding shows a higher burden of experiencing both diarrhea and respiratory infection symptoms than the previously done research. This could be because the current study mainly includes City inhabitants since City slum areas are often characterized by poor sanitation, overcrowding, and limited access to clean water and healthcare [27]. Furthermore, there is a difference in sociodemographic characteristics, and environmental factors such as climate and geographical differences because most of them are based on country-wide surveys [28].

Finally, a recall period of 2 weeks for diarrhea and 12 months for respiratory infection symptoms would lead the child to experience an enormous amount of respiratory infection symptoms which increases the chance of the child having comorbidity. The prevalence of diarrhea and respiratory infection symptoms was 24.28% and 35.29% respectively. Diarrhea and respiratory infection symptoms prevalence were comparable with the study conducted in eastern and northern Ethiopia at 22.5% and 22.1% [29, 30] and the study conducted in Gondar City at 37.5% respectively [31].

The prevalence of diarrhea was lower in comparison to a study conducted in the Southern part of Ethiopia 30.5% [32] and Northern part of Ethiopia almost half or 54% [33] of under-five children have diarrhea and higher in comparison to the studies conducted in other Ethiopia regions kamashi, farta and Addis Ababa 14.5%,16.7% and 11.9% respectively [34–36]. The prevalence of respiratory infection symptoms was lower in comparison to the studies conducted in India [37, 38] and higher than the study conducted previously in Gondar University Hospital pediatrics ward and Addis Ababa consecutively [11, 39].

We observed that children whose mothers/caretakers had no education their children more likely they have both conditions many findings also suggest this [2, 10, 25, 32, 40]. This is most likely due to a combination of circumstances like mothers/caretakers who had no formal education may be unaware of how to prevent diarrhea and ARI. For example, she/he is unaware of the significance of nursing, basic hygiene, and vaccination.

Children whose mothers/caretakers age between < 25 years are more likely to have morbidity of the two conditions than 25 years and above which is consistent with the finding from Kenya, Iran, and Nigeria [2, 25, 41]. This is due to the reason that older mothers/caretakers have more experience in preventing and managing childhood diseases and taking responsibility than the younger ones for their children. In addition, young women are believed to be fresh to childcare methods and hence lack such experience.

Children with second birth order are significantly associated with both conditions. Studies also showed that diarrhea occurrence is related to their birth order [42, 43]. Possible explanations for this association, first, later-born children may not receive as much attention from their parents, which could lead to poorer health outcomes. Secondly, later-born children may be less likely to be breastfed, which is protective against diarrhea and ARI. Children who started supplementary feeding greater than six months high likely to develop these conditions than those who started at 6 months and below, this finding also supported by Feachem published in the Bulletin of the World Health Organization reviews the evidence on the effectiveness of supplementary feeding programs in reducing the incidence and severity of diarrhea diseases in young children [44].

The explanation for this is starting supplementary feeding at 6 months can Improve the immune system and reduce the risk of malnutrition which will lead to a lower chance of getting diarrhea and respiratory infection symptoms than their counterparts. Children with playgrounds not clean have odds of comorbidity much higher than those who have clean playgrounds. This is because filthy surfaces contain infectious germs and this is corroborated by studies that found that poor sanitation and hygiene are linked to intestinal protozoa infections and diarrhea in those under-five in northern Ethiopia, it was also supported by findings from Bangladesh and Côte d’Ivoire [20, 45, 46].

The odds of comorbidity were higher among households that have a family size greater than five and this was consistent with studies from Uganda and Tanzania [47, 48]. The main reason for this is that larger families are more likely to live in crowded conditions, which can increase the risk of infection. Additionally, larger families may have fewer resources to invest in preventive measures, such as hand washing and access to clean water.

Mothers/caretakers who didn’t wash their hands after visiting the latrine and those who didn’t wash their hands with soap and water regularly their children were associated with higher odds of experiencing both diarrhea and respiratory infection symptoms. The explanation for this is washing your hands properly with soap and water can help to prevent the spread of germs (like bacteria and viruses) and can help to break the chain of infection and prevent the spread of germs that cause these diseases.

Other findings from developing countries like Bangladesh, Benin, Burkina, Faso Cambodia, Ghana, India, and Kenya also claim that hand hygiene interventions can reduce the incidence of diarrhea by 23% to 48% and the incidence of respiratory infections by 15% to 35% in schoolchildren in developing countries [49]. Studies from the University of Gondar Comprehensive Specialized Hospital and Lao People’s Democratic Republic supported this evidence stating that effective hand washing can prevent both diarrhea and ARI incidence [39, 50].

Even though this study did not find a significant relationship between income level breastfeeding status, kitchen floor, latrine availability, ownership of a latrine, the number of under-five, waste disposal method, and water source, other studies found that there is a significant association between getting sick for children with diarrhea and ARI with these factors [22, 42, 51–53].

## Conclusion

In this study, the prevalence of diarrhea and respiratory infection symptoms was relatively high in Gondar City. The independent predictors for comorbidity of diarrhea and respiratory infection symptoms are the mother/caretaker’s age, mother/caretaker’s education, child age, birth order, family size, supplementary feeding, and child playground, kitchen cleanses and hand hygiene practice. Preventive measures targeting both conditions simultaneously offer significant healthcare benefits for under-five populations.

## Data Availability

The datasets generated during and/or analyzed during the current study are available from the corresponding author upon reasonable request.

## Abbreviations

ARI: Acute respiratory infection
AOR: Adjusted odds ratio
CI: Confidence Interval
COR: Crude odds ratio
DHS: Demographic and Health survey
EDHS: Ethiopia Demographic and Health Survey
Epi-Info: Epidemiological information software
HIV: Human immune deficiency virus
IAP: Indoor air pollution
STATA: Statistics and data

## References

[1] Fenn B, Morris SS, Black RE. Comorbidity in childhood in northern Ghana: magnitude, associated factors, and impact on mortality. Int J Epidemiol. 2005;34(2):368–75. 15764695 10.1093/ije/dyh335

[2] Mulatya DM, Mutuku FW. Assessing comorbidity of diarrhea and acute respiratory infections in children under 5 years: evidence from Kenya’s demographic health survey 2014. J Prim Care Community Health. 2020;11:2150132720925190. 32450734 10.1177/2150132720925190 PMC7252376

[3] Rahman A, Hossain MM. Prevalence and determinants of fever, ARI and diarrhea among children aged 6–59 months in Bangladesh. BMC Pediatr. 2022;22(1):117. 35248016 10.1186/s12887-022-03166-9 PMC8897933

[4] Fenta SM, Nigussie TZ. Factors associated with childhood diarrheal in Ethiopia; a multilevel analysis. Archives of Public Health. 2021;79(1):1–12. 34229765 10.1186/s13690-021-00566-8 PMC8259006

[5] Liu L, Oza S, Hogan D, Perin J, Rudan I, Lawn JE, et al. Global, regional, and national causes of child mortality in 2000–13, with projections to inform post-2015 priorities: an updated systematic analysis. The lancet. 2015;385(9966):430–40. 10.1016/S0140-6736(14)61698-6 25280870

[6] Fullerton DG, Bruce N, Gordon SB. Indoor air pollution from biomass fuel smoke is a major health concern in the developing world. Trans R Soc Trop Med Hyg. 2008;102(9):843–51. 18639310 10.1016/j.trstmh.2008.05.028 PMC2568866

[7] Torres-Duque C, Maldonado D, Pérez-Padilla R, Ezzati M, Viegi G. Biomass fuels and respiratory diseases: a review of the evidence. Proc Am Thorac Soc. 2008;5(5):577–90. 18625750 10.1513/pats.200707-100RP

[8] Andualem Z, Azene ZN, Dessie A, Dagne H, Dagnew B. Acute respiratory infections among under-five children from households using biomass fuel in Ethiopia: systematic review and meta-analysis. Multidiscip Respir Med. 2020;15(1):710. 33437475 10.4081/mrm.2020.710 PMC7789869

[9] Islam T, Gauderman WJ, Berhane K, McConnell R, Avol E, Peters JM, et al. Relationship between air pollution, lung function and asthma in adolescents. Thorax. 2007;62(11):957–63. 17517830 10.1136/thx.2007.078964 PMC2117135

[10] Bokoro TA, Gebresilassie HK, Zeru MA. Joint binary response modelling for childhood comorbidity in Ethiopia. PLoS ONE. 2022;17(5): e0268040. 35584190 10.1371/journal.pone.0268040 PMC9116622

[11] Sanbata H, Asfaw A, Kumie A. Association of biomass fuel use with acute respiratory infections among under-five children in a slum urban of Addis Ababa. Ethiopia BMC public health. 2014;14(1):1–8. 10.1186/1471-2458-14-1122 PMC4237768 25358245

[12] Central Statistical Agency: Ethiopia Demographic and Health Survey; Ethiopia Demographic and Health Survey 2016 [cited 2023 21]. Available from: https://dhsprogram.com/publications/publication-fr328-dhs-final-reports.cfm.

[13] Schmidt W-P, Cairncross S, Barreto ML, Clasen T, Genser B. Recent diarrhoeal illness and risk of lower respiratory infections in children under the age of 5 years. Int J Epidemiol. 2009;38(3):766–72. 19279073 10.1093/ije/dyp159 PMC2689396

[14] Mulatu T, Yimer NB, Alemnew B, Linger M, Liben ML. Exclusive breastfeeding lowers the odds of childhood diarrhea and other medical conditions: evidence from the 2016 Ethiopian demographic and health survey. Ital J Pediatr. 2021;47(1):1–6. 34344434 10.1186/s13052-021-01115-3 PMC8335997

[15] gondar city population 2021 [cited 2023 23]. Available from: https://en.wikipedia.org/wiki/Gondar.

[16] Mekuriaw Alemayehu KA, Sharma HR, Gizaw Z, Shibru A. Household fuel use and acute respiratory infections in children under five years of age in Gondar city of Ethiopia. 2014.

[17] Andualem Z, Azene ZN, Azanaw J, Taddese AA, Dagne H. Acute respiratory symptoms and its associated factors among mothers who have under five-years-old children in northwest. Ethiopia Environmental Health and Preventive Medicine. 2020;25(1):1–12. 10.1186/s12199-020-00859-4 PMC7296770 32539699

[18] Natnael T, Lingerew M, Adane M. Prevalence of acute diarrhea and associated factors among children under five in semi-urban areas of northeastern Ethiopia. BMC Pediatr. 2021;21(1):290. 34174851 10.1186/s12887-021-02762-5 PMC8235618

[19] Dagnew AB, Tewabe T, Miskir Y, Eshetu T, Kefelegn W, Zerihun K, et al. Prevalence of diarrhea and associated factors among under-five children in Bahir Dar city, Northwest Ethiopia, 2016: a cross-sectional study. BMC Infect Dis. 2019;19:1–7. 31088387 10.1186/s12879-019-4030-3 PMC6518740

[20] Mekonnen HS, Ekubagewargies DT. Prevalence and factors associated with intestinal parasites among under-five children attending Woreta Health Center. Northwest Ethiopia BMC infectious diseases. 2019;19(1):1–8. 10.1186/s12879-019-3884-8 PMC6417120 30866833

[21] Anteneh ZA, Hassen HY. Determinants of Acute Respiratory Infection Among Children in Ethiopia: A Multilevel Analysis from Ethiopian Demographic and Health Survey. Int J Gen Med. 2020;13:17–26. 32099446 10.2147/IJGM.S233782 PMC6996624

[22] World Health Organization. Diarrhoeal disease Key facts 2017 [cited 2023 28]. Available from: https://www.who.int/news-room/fact-sheets/detail/diarrhoeal-disease.

[23] Manunâ MF, Nkulu-wa-Ngoie C. Factors associated with childs comorbid diarrhea and pneumonia in rural Democratic Republic of the Congo. African Journal of Medical and Health Sciences. 2020;19(5):55–62.

[24] Afrifa-Anane GF, Kyei-Arthur F, Agyekum MW, Afrifa-Anane EK. Factors associated with comorbidity of diarrhoea and acute respiratory infections among children under five years in Ghana. PLoS ONE. 2022;17(7): e0271685. 35862358 10.1371/journal.pone.0271685 PMC9302777

[25] Adedokun ST. Correlates of childhood morbidity in Nigeria: Evidence from ordinal analysis of cross-sectional data. PLoS ONE. 2020;15(5): e0233259. 32407377 10.1371/journal.pone.0233259 PMC7224558

[26] Myint1. SLT, W1. K, A1. KMP, YM3 A. Estimation of Acute Diarrhea and Acute Respiratory Infections among Children under Five Years Who Lived in a Peri-urban Environment of Myanma. OSIR. 2013;6:(4).

[27] Obasohan PE, Walters SJ, Jacques R, Khatab K. Risk Factors Associated with Multimorbidity among Children Aged Under-Five Years in Sub-Saharan African Countries: A Scoping Review. Int J Environ Res Public Health. 2023;20(2):1377. 36674135 10.3390/ijerph20021377 PMC9859535

[28] Okari MT. Public Health Implications Of The Housing, Water And Sanitation Conditions In Kaburini Slum Of Kakamega Town. Kenya: University Of Nairobi; 2019.

[29] Hossain MZ, Bambrick H, Wraith D, Tong S, Khan AF, Hore SK, et al. Sociodemographic, climatic variability and lower respiratory tract infections: a systematic literature review. Int J Biometeorol. 2019;63:209–19. 30680618 10.1007/s00484-018-01654-1

[30] Mengistie B, Berhane Y, Worku A. Prevalence of diarrhea and associated risk factors among children under-five years of age in Eastern Ethiopia: A cross-sectional study. Open J Prev Med. 2013;3(07):446.

[31] Getachew A, Tadie A, G Hiwot M, Guadu T, Haile D, G Cherkos T, et al. Environmental factors of diarrhea prevalence among under five children in rural area of North Gondar zone, Ethiopia. Ital J Pediatr. 2018;44(1):1–7. 10.1186/s13052-018-0540-7 PMC6097321 30115077

[32] Andualem Z, Taddese AA, Azene ZN, Azanaw J, Dagne H. Respiratory symptoms and associated risk factors among under-five children in Northwest, Ethiopia: community based cross-sectional study. Multidiscip Respir Med. 2020;15(1):685. 33117532 10.4081/mrm.2020.685 PMC7542992

[33] Mohammed S, Tamiru D. The burden of diarrheal diseases among children under five years of age in Arba Minch District, southern Ethiopia, and associated risk factors: a cross-sectional study. Int Sch Res Not. 2014;2014:654901. 10.1155/2014/654901 PMC4897213 27433486

[34] Berhe H, Mihret A, Yitayih G. Prevalence of diarrhea and associated factors among children under-five years of age in enderta woreda, tigray, northern ethiopia, 2014. International Journal of Therapeutic Applications. 2016;31:32–7.

[35] Fenta A, Alemu K, Angaw DA. Prevalence and associated factors of acute diarrhea among under-five children in Kamashi district, western Ethiopia: community-based study. BMC Pediatr. 2020;20:1–7. 32429989 10.1186/s12887-020-02138-1 PMC7236964

[36] Gedamu G, Kumie A, Haftu D. Magnitude and associated factors of diarrhea among under five children in Farta wereda. North West Ethiopia Qual Prim Care. 2017;25(4):199–207.

[37] Adane M, Mengistie B, Kloos H, Medhin G, Mulat W. Sanitation facilities, hygienic conditions, and prevalence of acute diarrhea among under-five children in slums of Addis Ababa, Ethiopia: Baseline survey of a longitudinal study. PLoS ONE. 2017;12(8): e0182783. 28854200 10.1371/journal.pone.0182783 PMC5576656

[38] Kumar SG, Majumdar A, Kumar V, Naik BN, Selvaraj K, Balajee K. Prevalence of acute respiratory infection among under-five children in urban and rural areas of puducherry, India. J Nat Sci Biol Med. 2015;6(1):3. 25810626 10.4103/0976-9668.149069 PMC4367064

[39] Savitha A, Gopalakrishnan S. Determinants of acute respiratory infections among under five children in a rural area of Tamil Nadu, India. Journal of family medicine and primary care. 2018;7(6):1268. 10.4103/jfmpc.jfmpc_131_18 PMC6293935 30613509

[40] Dagne H, Andualem Z, Dagnew B, Taddese AA. Acute respiratory infection and its associated factors among children under-five years attending pediatrics ward at University of Gondar Comprehensive Specialized Hospital, Northwest Ethiopia: institution-based cross-sectional study. BMC Pediatr. 2020;20:1–7. 32111196 10.1186/s12887-020-1997-2 PMC7047350

[41] Rahman A, Hossain MM. Prevalence and determinants of fever, ARI and diarrhea among children aged 6–59 months in Bangladesh. BMC Pediatr. 2022;22(1):1–12. 35248016 10.1186/s12887-022-03166-9 PMC8897933

[42] Ghasemi AA, Talebian A, Masoudi Alavi N, Moosavi G. Knowledge of mothers in management of diarrhea in under-five children, in kashan, iran. Nurs midwifery stud. 2013;1(3):158–62.

[43] Hashi A, Kumie A, Gasana J. Prevalence of diarrhoea and associated factors among under-five children in Jigjiga District, Somali Region, Eastern Ethiopia. Open J Prev Med. 2016;6(10):233–46.

[44] Mondal D, Paul P. Effects of indoor pollution on acute respiratory infections among under-five children in India: Evidence from a nationally representative population-based study. PLoS ONE. 2020;15(8):e0237611. 32797105 10.1371/journal.pone.0237611 PMC7428171

[45] Feachem RG. Interventions for the control of diarrhoeal diseases among young children: supplementary feeding programmes. Bull World Health Organ. 1983;61(6):967. 6609013 PMC2536232

[46] Ahmed NU, Zeitlin MF, Beiser AS, Super CM, Gershoff SN, Ahmed MA. Assessment of the impact of a hygiene intervention on environmental sanitation, childhood diarrhoea, and the growth of children in rural Bangladesh. Food Nutr Bull. 1994;15(1):40–52.

[47] Schmidlin T, Hürlimann E, Silué KD, Yapi RB, Houngbedji C, Kouadio BA, et al. Effects of hygiene and defecation behavior on helminths and intestinal protozoa infections in Taabo, Côte d’Ivoire. PLoS ONE. 2013;8(6):e65722. 23840358 10.1371/journal.pone.0065722 PMC3688730

[48] Omona S, Malinga GM, Opoke R, Openy G, Opiro R. Prevalence of diarrhoea and associated risk factors among children under five years old in Pader District, northern Uganda. BMC Infect Dis. 2020;20:1–9. 10.1186/s12879-020-4770-0 PMC6958783 31931735

[49] Sembua CLF. Determinants of recurrent diarrhea disease among children of under-five years residing in Tandale ward: Muhimbili University of Health and Allied Sciences; 2017.

[50] Mbakaya BC, Lee PH, Lee RL. Hand hygiene intervention strategies to reduce diarrhoea and respiratory infections among schoolchildren in developing countries: a systematic review. Int J Environ Res Public Health. 2017;14(4):371. 28368323 10.3390/ijerph14040371 PMC5409572

[51] Noguchi Y, Nonaka D, Kounnavong S, Kobayashi J. Effects of hand-washing facilities with water and soap on diarrhea incidence among children under five years in lao People’s Democratic Republic: A cross-sectional study. Int J Environ Res Public Health. 2021;18(2):687. 33466953 10.3390/ijerph18020687 PMC7829977

[52] Alambo KA. The prevalence of diarrheal disease in under five children and associated risk factors in Wolitta Soddo Town, Southern, Ethiopia. ABC Res Alert. 2015;3(2):13-22. Ethiopia-Ethiopia.

[53] Lamberti LM, Fischer Walker CL, Noiman A, Victora C, Black RE. Breastfeeding and the risk for diarrhea morbidity and mortality. BMC Public Health. 2011;11:1–12. 21501432 10.1186/1471-2458-11-S3-S15 PMC3231888

[54] Apanga PA, Kumbeni MT. Factors associated with diarrhoea and acute respiratory infection in children under-5 years old in Ghana: an analysis of a national cross-sectional survey. BMC Pediatr. 2021;21(1):1–8. 33581716 10.1186/s12887-021-02546-x PMC7881472

